# Effect of SGLT2 Inhibitors on Atrial Fibrillation in Patients With Type 2 Diabetes With Dilated Cardiomyopathy: A Cohort Study

**DOI:** 10.1155/cdr/5113761

**Published:** 2026-02-15

**Authors:** Jie Wang, Shanshan Qi, Siyuan Chen, Hang Yu

**Affiliations:** ^1^ Department of Cardiovascular Medicine, The First Affiliated Hospital of Xi′an Jiaotong University, Xi’an, China, xjtu.edu.cn; ^2^ Department of Health Medicine, The First Affiliated Hospital of Xi′an Jiaotong University, Xi’an, China, xjtu.edu.cn; ^3^ Department of Cardiovascular Surgery, The First Affiliated Hospital of Xi′an Jiaotong University, Xi’an, China, xjtu.edu.cn

**Keywords:** atrial fibrillation, cohort study, dilated cardiomyopathy, sodium-glucose cotransporter 2 inhibitors, Type 2 diabetes mellitus

## Abstract

**Background:**

Dilated cardiomyopathy (DCM) is characterized by left ventricular dilation and systolic dysfunction in the absence of severe hypertension, valvular disease, or coronary artery disease. Patients with DCM have a high risk of atrial fibrillation (AF), especially when combined with Type 2 diabetes mellitus (T2DM). Sodium‐glucose cotransporter 2 inhibitors (SGLT2i) have shown cardioprotective effects in patients with diabetes, suggesting potential benefits in reducing AF incidence.

**Methods:**

We retrospectively analyzed clinical data from patients with T2DM diagnosed with DCM treated at the First Affiliated Hospital of Xi’an Jiaotong University between January 2018 and January 2024. Patients were divided into two groups based on their use of SGLT2i. The incidence of AF was compared between these groups using logistic regression models adjusted for potential confounders, including demographic characteristics, comorbidities, and medication history. Additionally, a subgroup analysis was conducted to evaluate the relationship between AF and SGLT2i.

**Results:**

Among 455 enrolled patients, 95 (20.9%) were treated with SGLT2i. The incidence of AF was significantly lower in the SGLT2i group compared with the control group (7/95 [7.4%] vs. 68/360 [18.9%]; OR = 0.308, 95*%*CI = 0.132–0.735, *p* = 0.008). Subgroup analyses showed consistent results across various age, gender, hypertension status, and diabetes duration groups, indicating the robustness of the association between SGLT2i use and reduced AF incidence.

**Conclusion:**

Our study suggests that SGLT2i use is associated with a lower incidence of AF in patients with T2DM and DCM. This observed association warrants further investigation in prospective studies to elucidate its nature.

## 1. Introduction

Dilated cardiomyopathy (DCM) is characterized by the enlargement of the left ventricle and impaired systolic function, manifesting in the absence of abnormal loading conditions or notable coronary artery disease [1]. The prevalence of DCM was 40 cases per 100,000 people, and the annual incidence is seven cases per 100,000 people [2]. The most common symptoms are related to congestive heart failure, while circulatory failure, arrhythmias, and thromboembolic events also frequently observed in these patients [3]. Previous studies have found a 25%–30% mortality rate within the first year post‐diagnosis, and a 50% survival rate at 5 years [4]. Previous studies have found that patients with DCM always exhibit an elevated risk of developing atrial fibrillation (AF), which was associated with poor prognosis [5].

Chronic hyperglycemia induces progressive damage, dysfunction, and eventual failure of multiple organs. Patients with diabetes often have comorbidities such as obesity, hypercholesterolemia, atherosclerosis, microcirculation disorders, and hypertension, significantly increasing the risk of cardiac damage, including cardiomyopathy, coronary artery disease, and AF [6]. Notably, chronic low‐grade inflammation serves as a central pathogenic mechanism linking these metabolic and cardiovascular disorders, promoting endothelial dysfunction, atherosclerosis, and arrhythmogenesis, thereby contributing to worse clinical outcomes in patients with CVDs [7].

Cardiovascular complications are the leading cause of death in patients with diabetes. Although most cardiovascular events in patients with diabetes are caused by atherosclerosis, a significant portion is also due to embolism related to AF. AF is the most common cardiac arrhythmia, with increased incidence and prevalence in Type 2 diabetes [8].

Sodium‐glucose cotransporter 2 inhibitors (SGLT2i) are a class of glucose‐lowering drugs that reduce blood glucose by inhibiting glucose reabsorption in the proximal renal tubules. This class includes drugs such as dapagliflozin and empagliflozin, which not only have glucose‐lowering effects but also provide significant protective benefits for heart and kidney function [9]. Several mechanistic studies propose that the association between SGLT2i and lower arrhythmia risk may be explained by improvements in myocardial energy metabolism, inhibiting inflammation, reducing oxidative stress, and ameliorating atrial remodeling [10]. Consistent with these potential mechanisms, some clinical studies and meta‐analyses have reported a lower incidence of AF among SGLT2i users [11]. However, the evidence remains inconsistent. Notably, findings from large randomized controlled trials and meta‐analyses have been conflicting [12, 13]; for instance, the EMPA‐REG OUTCOME trial suggested a potential increase in AF incidence with empagliflozin, whereas the DECLARE‐TIMI 58 trial reported a neutral or potentially beneficial effect of dapagliflozin on AF risk [14, 15]. This discrepancy underscores the complexity of the issue and the critical lack of prospective evidence in certain clinical settings.

However, growing evidence points to the direct modulatory effects of SGLT2i on cardiac electrophysiology and the autonomic nervous system as a plausible explanation for the observed association with lower arrhythmia incidence [16]. Specifically, a pivotal clinical study demonstrated that SGLT2i therapy in patients with Type 2 diabetes significantly improved cardiac autonomic function, quantified by heart rate variability and myocardial scintigraphy, and this improvement was directly associated with a reduced incidence of vasovagal syncope, a neurally mediated arrhythmic event [17]. This suggests that the potential antiarrhythmic properties of SGLT2i may extend to AF through the stabilization of neuro‐cardiac interplay. However, the evidence regarding this specific effect in high‐risk populations with coexisting T2DM and DCM remains scarce.

Importantly, a recent prospective study specifically designed to evaluate the antiarrhythmic effects of SGLT2i in patients with diabetes with acute myocardial infarction has provided novel insights, demonstrating a significant reduction in in‐hospital arrhythmic burden [18]. This emerging evidence not only strengthens the rationale for investigating SGLT2i’s antiarrhythmic potential but also highlights the need to further explore their role in the acute phase of myocardial infarction, a period of heightened vulnerability to life‐threatening arrhythmias.

At the same time, most current studies on SGLT2i and AF risk have focused on broad populations of patients with heart failure or diabetes, while evidence specifically for the high‐risk population with both T2DM and DCM remains limited and inconclusive. This particular population endures the dual burden of metabolic disturbances and specific myocardial pathology, leading to an exceptionally high risk of AF development and poorer prognosis. Therefore, assessing the anti‐arrhythmic effect of SGLT2i in such patients is of significant clinical importance. Existing large randomized controlled trials (e.g., EMPA‐REG OUTCOME and DECLARE‐TIMI 58) have reported inconsistent findings regarding AF outcomes in this specific subgroup, and observational studies rarely focus exclusively on this cohort [14, 15]. This evidence gap justifies the need for further investigation.

Therefore, this study aims to retrospectively collect clinical data from patients with diabetes with DCM treated at our hospital, and investigate the effect of SGLT2i on the incidence of AF in these patients. We will analyze the incidence of AF in these patients to provide evidence‐based medical data for risk stratification and intervention in preventing arrhythmias in this patient population.

## 2. Materials and Methods

### 2.1. Population

We enrolled patients diagnosed with DCM and Type 2 diabetes who were treated at the First Affiliated Hospital of Xi’an Jiaotong University from January 2018 to January 2024. The inclusion criteria were as follows: (1) diagnosed with DCM; (2) diagnosed with Type 2 diabetes; (3) aged between 18 and 80 years. The diagnosis of DCM was confirmed following the current guidelines set by the European Society of Cardiology (ESC) guidelines, which include the following: 1) the detection of left ventricular (LV) dilation and reduced LV systolic function and 2) the exclusion of significant coronary artery disease (CAD), congenital heart disease, arterial hypertension, and primary valvular disease [19]. The diagnosis of T2DM was established according to the World Health Organization criteria, including the following: (1) glycated hemoglobin (HbA1c) levels ≥ 6.5%, (2) fasting plasma glucose levels ≥ 7.0 mmol/L, and (3) random plasma glucose levels ≥ 11.1 mmol/L accompanied by hyperglycemic symptoms [20].

**Figure 1:**
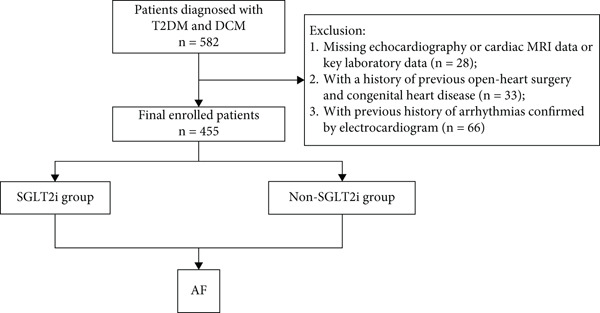
Study protocol. The flowchart of enrollment of study patients. Abbreviations: AF, atrial fibrillation; DCM, diabetic cardiomyopathy; T2DM, type 2 diabetes mellitus.

The exclusion criteria were as follows: (1) missing echocardiography or cardiac MRI data or key laboratory data; (2) with a history of previous open‐heart surgery and congenital heart disease; (3) with previous history of arrhythmias confirmed by electrocardiogram (Figure 1); (4) pre‐existing AF prior to the initiation of SGLT2i therapy. This study adhered to the Declaration of Helsinki and received approval from the Ethics Committee of Xi’an Jiaotong University (XJTU1AF2024LSYY‐99).

At admission, demographic data for all patients were collected, including gender, age, systolic blood pressure, and diastolic blood pressure. The comorbidities and laboratory data were collected from the medical record. Medication history comprised the use of angiotensin‐converting enzyme inhibitors (ACEIs), angiotensin II receptor blockers (ARBs), *β*‐blockers, and antidiabetic drugs, including insulin, metformin, DPP‐4 inhibitors, GLP‐1 receptor agonists (GLP‐1RAs), sulfonylureas, alpha‐glucosidase inhibitors, and glinides. The duration of diabetes was also documented, with the temporal relationship between SGLT2i initiation and AF diagnosis explicitly recorded to ensure that the incidence of AF was assessed only after the start of SGLT2i therapy.

### 2.2. Method of Grouping

The enrolled patients were divided into the SGLT2i group and the control group according to the use of SGLT2i. Patients in the SGLT2i group were defined as those with continuous SGLT2i use for at least 3 months prior to the index admission/enrollment. This duration was selected to ensure a biologically plausible exposure window, allowing sufficient time for the drug’s hemodynamic and metabolic effects to stabilize and translate into potential clinical benefits. Furthermore, a 3‐month threshold helps to mitigate the potential misclassification of exposure by distinguishing between transient and sustained use, thereby increasing the specificity of our exposure definition.

### 2.3. Clinical Outcomes

In this study, the clinical outcome of interest was AF, which was systematically evaluated at hospital admission and during each follow‐up visit. The diagnosis of AF according to the ESC guidelines is based on specific ECG findings. These include absolutely irregular RR intervals and the absence of distinct, identifiable P waves [21]. It is noteworthy that in this study, a systematic approach was adopted for AF detection: all patients underwent standardized 12‐lead ECG recording at admission and during follow‐up clinic visits. All ECG traces were independently reviewed and confirmed by two cardiologists to ensure consistent application of the diagnostic criteria. This systematic ECG assessment enhances the reliability of AF incidence estimation in our cohort.

### 2.4. Statistical Analysis

Continuous variables exhibiting a normal distribution were described as mean ± standard deviation while those were not normally distributed were expressed as median and interquartile range. The independent samples *t* test and Mann–Whitney *U* test were used to evaluate differences between two groups. The categorical variables were presented as counts and percentages, with comparisons between groups using the chi‐square test.

The association between SGLT2i use and AF was evaluated using univariate and multivariate logistic regression analyses. We constructed three logistic regression models to assess this relationship. Model 1 was unadjusted, including only the use of SGLT2i. Model 2 was adjusted for age and sex. For the fully adjusted Model 3, we adopted an inclusive approach by incorporating a comprehensive set of clinically relevant covariates known to be potential confounders, aiming to rigorously test the stability of the observed association. Accordingly, Model 3 was further adjusted for the following covariates: age; sex; admission systolic and diastolic blood pressure; cardiac function, quantified by the New York Heart Association (NYHA) classification; duration of diabetes, ascertained from medical records; and comorbidities including hypertension (diagnosed based on elevated admission blood pressure or documented history), hyperlipidemia (diagnosed by laboratory criteria), chronic obstructive pulmonary disease (COPD), and chronic kidney disease (CKD), with the latter two diagnosed according to established guidelines. Laboratory indicators adjusted for included HbA1c, NT‐proBNP, creatinine, low‐density lipoprotein (LDL), high‐density lipoprotein (HDL), and blood urea nitrogen (BUN) and medication history. Subgroup analyses were conducted to determine whether the association between SGLT2i use and the incidence of AF differed according to various population characteristics. Also, to address potential bias resulting from the exclusion of patients with missing critical data, we employed multiple imputation techniques. In our dataset, the variables with missing values were the following laboratory parameters: HbA1c, NT‐proBNP, creatinine (Cr), low‐density lipoprotein (LDL), high‐density lipoprotein (HDL), and blood urea nitrogen (BUN). The missingness mechanism for these variables was verified to be consistent with the Missing at Random (MAR) assumption. All statistical analyses were conducted using SPSS 27.0, with a two‐sided *p* < 0.05 deemed statistically significant.

## 3. Results

### 3.1. Baseline Characteristics

Among the initial 582 patients with DCM combined with T2DM enrolled for this study, 28 were missing critical data, 33 had congenital heart disease or a history of open‐heart surgery, and 66 had a prior history of arrhythmia. Ultimately, 455 consecutive patients were finally included in this study.

The baseline characteristics of patients were shown in Table 1. The age, proportion of male patients and the patients with poor cardiac function were comparable between the two groups (all *p* > 0.05). There was no statistically significant difference in systolic and diastolic blood pressure at admission between the two groups (all *p* > 0.05). Similarly, the duration of diabetes did not differ significantly between the groups (*p* > 0.05). Regarding comorbidities, there were no differences in the hypertension, COPD, and CKD between the groups (all *p* > 0.05). However, a higher percentage of patients in the SGLT2 inhibitor group had hyperlipidemia compared with the control group (8.42% vs. 3.06%, *p* = 0.042). Additionally, the SGLT2 inhibitor group exhibited higher HbA1c levels [7.50 (7.00, 8.20) vs. 7.10 (6.70, 7.80), *p* = 0.004], low‐density lipoprotein (LDL) [2.32 (1.94, 2.66) vs. 2.16 (1.81,2.50), *p* = 0.015], high‐density lipoprotein (HDL) [0.89 (0.82, 1.02) vs. 0.86 (0.76, 0.95), *p* = 0.002] and lower NT‐proBNP levels [1441.00 (818.00,3554.00) vs. 2308.00 (1281.75, 3810.00), *p* < 0.001]. Apart from a higher rate of insulin use in the control group (7.37% vs. 17.78%, *p* = 0.013), the usage rates of other antidiabetic medications, including Metformin, DPP‐4 inhibitors, GLP‐1 receptor agonists, sulfonylureas, *α*‐glucosidase inhibitors, and thiazolidinediones, did not differ significantly between the two groups (all *p* > 0.05).

**Table 1:** Clinical characteristics of patients with T2DM combined with DCM.

**Characteristics**	**SGLT2i group (** **N** = 95**)**	**Control group (** **N** = 360**)**	**p** **value**
Male (*n*, %)	68 (71.58)	263 (73.06)	0.774
Age (years)	58.00 (45.00, 64.00)	58.00 (49.00, 65.00)	0.390
SBP (mmHg)	124.00 (111.00, 136.00)	120.00 (113.00, 136.75)	0.988
DBP (mmHg)	83.00 (69.00, 88.00)	78.00 (72.00, 87.75)	0.670
NYHA > 2 (*n*, %)	59 (62.11)	254 (70.56)	0.114
Diabetes duration (years)	1.00 (0.00, 5.00)	3.00 (0.00, 7.75)	0.168
Other comorbidities, *n* (%)			
Hypertension	51 (53.68)	190 (52.78)	0.875
Hyperlipidemia	8 (8.42)	11 (3.06)	0.042
COPD	2 (2.11)	12 (3.33)	0.777
CKD	1 (1.05)	15 (4.17)	0.249
Laboratory variables			
HbA1bc (%)	7.50 (7.00, 8.20)	7.10 (6.70, 7.80)	0.004
NT‐proBNP	1441.00 (818.00, 3554.00)	2308.00 (1281.75, 3810.00)	< 0.001
Cr (umol/L)	73.00 (64.00, 85.00)	74.00 (60.00, 91.75)	0.550
LDL (mmol/L)	2.32 (1.94, 2.66)	2.16 (1.81, 2.50)	0.015
HDL (mmol/L)	0.89 (0.82, 1.02)	0.86 (0.76, 0.95)	0.002
BUN (mmol/L)	7.19 (5.78, 8.56)	6.96 (5.82, 8.85)	0.887
Medications			
ACEI/ARB	28 (29.47)	95 (26.39)	0.547
*β* blocker	23 (24.21)	67 (18.61)	0.223
Insulin	7 (7.37)	64 (17.78)	0.013
Metformin	29 (30.53)	103 (28.61)	0.714
DPP‐4	2 (2.11)	7 (1.94)	1.000
GLP‐1RAs	3 (3.16)	3 (0.83)	0.207
Sulfonylurea	6 (6.32)	36 (10.00)	0.270
*α*‐Glucosidase inhibitors	18 (18.95)	46 (12.78)	0.124
Glitazone	0 (0.00)	7 (1.94)	0.368

Abbreviations: ACEI, angiotensin‐converting enzyme inhibitors; ARB, angiotensin II receptor blockers; BUN, blood urea nitrogen; CKD, chronic kidney disease; COPD, chronic obstructive pulmonary disease; Cr, creatinine; DBP, diastolic blood pressure; DCM, diabetic cardiomyopathy; DPP‐4, dipeptidyl peptidase‐4 inhibitors; GLP‐1RA: glucagon‐like peptide‐1 receptor agonists; HDL, high‐density lipoprotein; LDL, low‐density lipoprotein; SBP, systolic blood pressure; T2DM, type 2 diabetes mellitus.

### 3.2. The Association of SGLT2i With AF in the Total Population

The results of multivariate logistic regression analysis model adjusted for confounding factors were shown in Table 2. Model 1, the unadjusted model, indicates a negative correlation between the use of SGLT2i and the occurrence of AF (OR = 0.342, 95*%*CI = 0.151–0.771, *p* = 0.010). In Model 2, this association remains stable after adjusting for age and gender (OR = 0.353, 95*%*CI = 0.156–0.800, *p* = 0.013). In the fully adjusted Model 3, the risk of AF in patients with DCM with Type 2 diabetes using SGLT2i is 0.312 times that of patients not using SGLT2i (OR = 0.308, 95*%*CI = 0.132–0.735, *p* = 0.008), suggesting that SGLT2i use was associated with a reduced risk of AF in this patient population.

**Table 2:** Logistic analysis for association of AF with SGLT2i in patients with T2DM combined with DCM.

	**OR**	**95% CI**	**p** **value**
Model 1	0.342	0.151–0.771	0.010
Model 2	0.353	0.156–0.800	0.013
Model 3	0.308	0.132–0.735	0.008

*Note:* Model 1, adjusted for none; Model 2, adjusted for age and sex; Model 3, adjusted for age, sex, admission systolic and diastolic blood pressure, cardiac function, duration of diabetes, hypertension, hyperlipidemia, chronic obstructive pulmonary disease, chronic kidney disease, laboratory variables and medication history.

Abbreviations: CI, confidence interval. OR, odds ratios. Other abbreviations as in Table 1.

### 3.3. Sensitivity Analyses and Interaction Analysis

After performing multiple imputation to address missing data, including the 28 patients previously excluded, we found that the results from the multivariate logistic regression analysis were consistent with the primary analysis. The use of SGLT2i was associated with a reduced risk of AF in patients with DCM and T2DM (Table 3). The interaction between SGLT2i and age was not statistically significant (*p* = 0.462), and the interaction between SGLT2i and diabetes duration was also not significant (*p* = 0.268). These findings suggest that the effect of SGLT2i on AF risk is consistent across different age groups and diabetes durations.

**Table 3:** The relationship between SGLT2i and AF, after applying multiple imputation to address missing data in covariates.

	**Participants (** **n** = 483**)**		
	**OR**	**95% CI**	**p** **value**
Model 1	0.418	0.208–0.842	0.015
Model 2	0.434	0.214–0.879	0.020
Model 3	0.308	0.130–0.732	0.008

*Note:* Model 1, adjusted for none; Model 2, adjusted for age and sex; Model 3, adjusted for age, sex, admission systolic and diastolic blood pressure, cardiac function, duration of diabetes, hypertension, hyperlipidemia, chronic obstructive pulmonary disease, chronic kidney disease, laboratory variables and medication history.

Abbreviations: CI, confidence interval; OR, odds ratios. Other abbreviations as in Table 1.

### 3.4. Stratification Analysis

**Figure 2:**
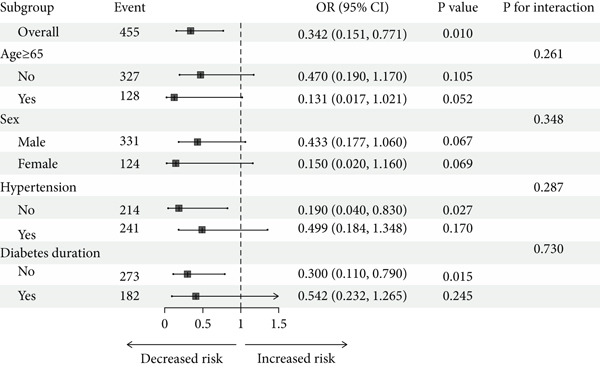
Stratified analysis of associations between AF and SGLT2i. The results were adjusted for all covariates except the corresponding stratification variable. Abbreviations: CI, confidence interval; OR, odds ratios.

The results of the subgroup analysis indicate that the therapeutic effects of SGLT2i vary among patients with different ages, genders, hypertension statuses, and durations of diabetes. Patients under 65 years old had a greater risk of AF (OR = 0.470, 95*%*CI = 0.190–1.170) compared with those over 65 (OR = 0.131, 95*%*CI = 0.017–1.021). However, these differences are not statistically significant (*p* for interaction > 0.05 for all subgroups), suggesting that the relationship between SGLT2i and AF is consistent across all subgroups (Figure 2).

## 4. Discussion

In this single‐center, retrospective cohort study, we investigated the association between SGLT2 inhibitor use and incident AF in a high‐risk population of patients with concomitant Type 2 diabetes mellitus and DCM. By analyzing data from 455 patients and employing multivariate logistic regression models to control for potential confounders, we found that treatment with SGLT2i was independently associated with a significantly lower incidence of AF compared with nonuse (7.4% vs. 18.9%; adjusted OR = 0.308). This association remained consistent across various patient subgroups, including those defined by age, gender, and hypertension status.

Our observation of a significant association between SGLT2i use and reduced AF incidence in this specific cohort offers a nuanced perspective against the backdrop of conflicting results from large RCTs and meta‐analyses [13]. We postulate several plausible explanations for this discrepancy. Foremost is the distinctiveness of our study population—patients with the dual pathophysiology of T2DM and DCM may represent a subgroup that derives exceptional benefit from the pleiotropic cardioprotective effects of SGLT2i. Additionally, methodological variations in endpoint ascertainment must be considered; our systematic review of all available ECGs might have captured AF episodes that were not reported as adverse events in trials. Notably, the lower baseline NT‐proBNP levels in the SGLT2i group, potentially an early marker of the drug’s hemodynamic effects, underscores a potent reversal of ventricular stress—a key arrhythmogenic mechanism in DCM that might be particularly relevant to the observed association with lower AF risk.

This potential reduction in AF risk carries substantial clinical importance. The development of AF in this vulnerable population often necessitates long‐term oral anticoagulation, which is a well‐established risk factor for major bleeding events. This is particularly concerning, as recent evidence continues to highlight the substantial bleeding risk faced by patients on long‐term anticoagulation [22]. Therefore, our finding suggests a potential dual benefit of SGLT2i: not only potentially directly reducing the arrhythmic burden but also indirectly mitigating the future risk of anticoagulation‐associated bleeding by potentially obviating the need for such therapy in a proportion of patients. This underscores the clinical relevance of our results, extending beyond the arrhythmia itself to impact overall patient safety and management.

Currently, with the application of SGLT2I in the treatment of T2DM, the impact of SGLT2i on the incidence of AF in patients with T2DM remains unclear based on evidence from large‐scale randomized clinical trials. In the EMPA‐REG OUTCOME trial, which investigated cardiovascular events in patients with T2DM, the incidence of new‐onset AF was higher in the empagliflozin group (2.3%) compared with the placebo group (1.6%) [23]. A cohort study involving 399,810 patients of T2DM revealed that the risk of AF in these patients prescribed SGLT‐2i was comparable with that in a 1:1 propensity score‐matched cohort of patients who did not receive SGLT2i therapy [24]. However, with the deepening of research, SGLT2i have been demonstrated to possess cardioprotective effects through various mechanisms, which may also contribute to a reduced risk of arrhythmias. A post‐hoc analysis of the DECLARE‐TIMI 58 trial indicates that dapagliflozin significantly reduces the relative risk of atrial arrhythmias [15]. Li et al. found that after adjusting for age, HbA1c, blood pressure, and weight, the association between SGLT2i and a reduced risk of AF remained consistent in patients with T2DM. Furthermore, this association was more pronounced with longer durations of SGLT2i treatment. They also have found that the incidence of heart failure in patients treated with SGLT2i is significantly lower than in those receiving placebo [25]. Because of the presence of common risk factors and closely related pathophysiological processes, AF and heart failure always interact with each other [26]. This finding was consistent with our research results, which indicate that patients in the SGLT2i group have lower NT‐proBNP levels compared with those not using SGLT2i. The apparent inconsistency between our findings (showing a benefit) and some prior trials could be attributed to several factors. Firstly, the study population is a key consideration. Our study specifically focused on a high‐risk cohort of patients with concomitant T2DM and DCM, who endure a dual burden of metabolic disturbance and specific myocardial pathology. This population exhibits a profoundly elevated risk for AF and may derive a disproportionately greater benefit from the multifaceted cardioprotective effects of SGLT2i compared with the broader populations enrolled in large cardiovascular outcome trials (CVOTs), which predominantly included patients with T2DM and established atherosclerotic cardiovascular disease or multiple risk factors, but not necessarily DCM. Second, differences in study design and endpoint ascertainment may contribute to the divergent results. Large RCTs like EMPA‐REG OUTCOME adjudicated AF as a prespecified adverse event, which might underreport its incidence compared with our retrospective study that systematically reviewed medical records and electrocardiographic data for AF diagnosis. Furthermore, variations in background therapy, follow‐up duration, and the specific SGLT2i agent used (e.g., empagliflozin vs. dapagliflozin) could also influence the outcomes. Our observation of lower NT‐proBNP levels in the SGLT2i group suggests a stronger effect on reversing volume overload and pressure stress in this DCM population, which might be a key mechanism underlying the observed association with lower AF risk in these patients.

These potential mechanisms have been further elucidated in basic research. For instance, previous studies have found that SGLT2i use has been associated with improvements in atrial structural and electrical remodeling in patients with Type 2 diabetes, and is linked to enhanced mitochondrial function and biogenesis, and may potentially be used for the prevention of T2DM‐associated AF [27, 28]. Also, the anti‐arrhythmic effects of SGLT2i have been attributed to several mechanisms observed in preclinical studies, including inhibition of the sodium–hydrogen exchange in cardiomyocytes, suppression of the sympathetic nervous system, and a reduction in the accumulation and inflammation of visceral and perivascular adipose tissues [29–31]. Increased glucose excretion leads to additional osmotic diuresis, which results in a reduction in arterial blood pressure and delays myocardial structural remodeling, consequently slowing the progression of atrial fibrosis [32]. On the other hand, the loss of glucose in the urine during diuresis may contribute to weight loss and long‐term weight maintenance, thereby reducing atrial dilation and the incidence of AF [33, 34].

Yin et al. have found that SGLT2i could reduce the risk of AF in patients with heart failure by enhancing mitochondrial function. This is achieved through increased expression of PGC‐1*α* and elevated ATP levels, which in turn decrease reactive oxygen species (ROS) production and stabilize myocardial cell membrane potential [35]. SGLT2 inhibitor enhances glycemic control and improves insulin sensitivity, which can reduce the adverse effects of hyperglycemia and hyperinsulinemia on cardiac electrophysiology. Also, SGLT2i may improve sodium and calcium handling abnormalities in atrial cardiomyocytes associated with HFrEF and AF, thereby preventing the initiation and maintenance of AF [36]. SGLT2i treatment is associated with improved renal function, which may indirectly lead to enhanced cardiac function by reducing afferent sympathetic nervous system activation, alleviating inflammation, and improving oxidative stress [37]. SGLT2i treatment has been correlated with a reduction in the expression of myocardial fibrosis markers, which could potentially mitigate myocardial fibrosis and lead to improvements in myocardial structure, which is crucial for the prevention of AF. Improvements in left ventricular ejection fraction associated with SGLT2i may contribute to enhanced cardiac pump function and attenuated ventricular remodeling, thereby potentially improving overall cardiac function. Moreover, SGLT2i markedly decrease oxidative stress levels and inflammatory responses within the myocardium. These factors play a critical role in the development of heart failure and AF, and their reduction helps protect myocardial cells, thereby lowering the risk of AF. SGLT2i reduce cardiac stress and burden by promoting natriuresis and diuresis, which in turn decreases blood volume and cardiac preload. Additionally, SGLT2i improve glycemic control and overall metabolic status, including reducing lipid levels and enhancing insulin sensitivity, which further promotes cardiac health [38].

Current treatments for AF include both pharmacological and non‐pharmacological interventions. Beta‐blockers and calcium channel blockers are commonly used for ventricular rate control, while antiarrhythmic drugs help maintain sinus rhythm [39]. However, these medications often come with side effects, for instance, amiodarone is associated with thyroid and pulmonary complications, raising safety concerns with long‐term use. In terms of non‐pharmacological strategies, catheter ablation is a key option for refractory patients with AF [12]. Nevertheless, the efficacy of this procedure may decline over time, and its effectiveness can be limited in patients with comorbid conditions such as diabetes or heart failure. In contrast, SGLT2i offer not only glycemic control but also significant cardioprotective benefits. Compared with traditional therapies, SGLT2i reduce AF risk by mitigating oxidative stress and inflammation, improving cardiac remodeling, and enhancing metabolic status. Their favorable safety profile and additional cardiovascular benefits make them particularly advantageous for patients with both diabetes and AF.

The results of our subgroup analysis are consistent with those of the overall population analysis. Across subgroups defined by age, gender, hypertension status, and diabetes duration, a trend of lower AF incidence was consistently observed in patients using SGLT2 inhibitors compared with nonusers. The consistency of this association across diverse patient characteristics may indicate that the potential cardiovascular benefits of SGLT2 inhibitors extend to a broad range of patients, though this observation requires confirmation in future studies.

We acknowledge the potential for confounding by standard heart failure medications. However, several aspects of our data suggest that the observed association between SGLT2i and reduced AF incidence is independent of such therapies. As detailed in Table 1, the baseline use of foundational heart failure treatments, including ACEIs/ARBs and *β*‐blockers, was well‐balanced between the SGLT2i and control groups, with no statistically significant differences. Crucially, our multivariate logistic regression models (Model 3) explicitly adjusted for the use of these very medications, and the association between SGLT2i and reduced AF risk remained robust and statistically significant (OR = 0.308, *p* = 0.008). This indicates that the beneficial effect of SGLT2i is not merely a surrogate for more intensive background heart failure therapy but likely represents an independent or additive protective effect against atrial arrhythmogenesis in this high‐risk population.

However, the interpretation of the observed association between SGLT2 inhibitor use and reduced AF incidence requires careful consideration of baseline imbalances between the groups. As shown in Table 1, patients in the SGLT2i group exhibited a distinct clinical profile at baseline, characterized by higher HbA1c and LDL levels, lower NT‐proBNP levels, and lower insulin use compared with the control group. This profile suggests potentially poorer baseline glycemic and lipid control but possibly less severe heart failure in the SGLT2i group. It is important to consider that some of these differences, particularly in metabolic parameters (HbA1c, LDL), might not solely represent confounding but could also be early markers of the hemodynamic and metabolic effects of SGLT2i therapy (e.g., volume depletion, metabolic shift) that had initiated prior to outcome assessment. Although we rigorously adjusted for these and other potential confounders in our multivariable logistic regression models (Model 3), the possibility of residual confounding from unmeasured factors (e.g., intensity of care, socioeconomic status) persists. Nonetheless, the association between SGLT2i use and lower AF incidence remained following comprehensive adjustment. When considered alongside the biologically plausible explanation that some baseline imbalances may reflect early drug action, this finding provides support for a potential relationship between SGLT2i and reduced AF risk in this patient population.

We acknowledge several limitations in this study, each of which illuminates a pathway for future scientific inquiry. It is important to note that the non‐randomized, observational design, while suitable for generating hypotheses in this understudied population, inherently prevents definitive causal conclusions due to residual confounding. Consequently, our findings should be interpreted as hypothesis‐generating, warranting validation in dedicated randomized controlled trials specifically enrolling patients with T2DM and DCM to provide the highest level of evidence. Furthermore, our assessment of AF was reliant on standard 12‐lead ECGs and documented history, which precluded the analysis of AF subtypes and overall arrhythmic burden. Future prospective studies should therefore incorporate prolonged cardiac monitoring to precisely quantify AF burden and differentiate the effect of SGLT2i across paroxysmal and persistent subtypes. Another consideration is that our definition of SGLT2i use, though ensuring a minimum exposure window, lacked granularity regarding specific agent, dosage, and patient adherence. Subsequent pharmaco‐epidemiological studies leveraging large, detailed prescription databases could explore potential dose‐response relationships and comparative effectiveness between different SGLT2 inhibitors. Finally, given the single‐center nature of our study, the generalizability of our findings may be limited. Future multicenter, international collaborations are needed to confirm this association across diverse healthcare settings and ethnic populations. Additionally, the discussion in our study is inherently limited by the available data and does not address the longitudinal progression or the clinical severity of AF episodes. Future investigations with detailed temporal data are encouraged to explore the dynamic impact of SGLT2i on AF burden over time.

Despite these limitations, our study provides robust preliminary evidence for a strong association between SGLT2i use and reduced AF incidence in a high‐risk, targeted population. The limitations discussed here do not diminish our findings but, rather, chart a clear and necessary course for the next stages of investigation in this field.

## 5. Conclusion

In summary, the use of SGLT2 inhibitors was associated with a significantly lower incidence of AF in patients with DCM and T2DM. These findings provide preliminary evidence supporting a potential association between SGLT2i use and lower arrhythmia incidence within this high‐risk population. If confirmed by prospective randomized trials, this finding could have important implications for the management of this high‐risk population.

**Nomenclature**
ACEIs: Angiotensin‐converting enzyme inhibitors
AF: Atrial fibrillation
ARBs: Angiotensin II receptor blockers
CAD: Coronary artery disease
CKD: Chronic kidney disease
COPD: Chronic obstructive pulmonary disease
DCM: dilated cardiomyopathy
ESC: European Society of Cardiology
GLP‐1Ras: GLP‐1 receptor agonists
HbA1c: Glycated hemoglobin
HDL: High‐density lipoprotein
HFrEF: Heart failure with reduced ejection fraction
IQR: Interquartile range
LDL: Low‐density lipoprotein
LV: Left ventricular
SD: Standard deviation
SGLT2i: Sodium‐glucose cotransporter 2 inhibitors
T2DM: Type 2 diabetes mellitus

## Ethics Statement

This study adhered to the Declaration of Helsinki and received approval from the Ethics Committee of Xi′an Jiaotong University (XJTU1AF2024LSYY‐99).

## Consent

The authors have nothing to report.

## Disclosure

All authors have read and agreed to the published version of the manuscript.

## Conflicts of Interest

The authors declare no conflicts of interest.

## Author Contributions

Conceptualization, Siyuan Chen and Shanshan Qi; methodology, Siyuan Chen, Shanshan Qi, and Jie Wang; software, Siyuan Chen, Shanshan Qi, and Jie Wang; validation, Siyuan Chen and Shanshan Qi; investigation, Siyuan Chen, Shanshan Qi, and Jie Wang; writing – original draft preparation, Siyuan Chen, Shanshan Qi, Jie Wang, and Hang Yu; visualization, Siyuan Chen, Shanshan Qi, Jie Wang, and Hang Yu; supervision, Hang Yu. Jie Wang and Shanshan Qi contributed equally to this work and shared the first authorship.

## Funding

The study was supported by the Key Project of Research and Development Plan of Shaanxi Province, China (2025SF‐YBXM‐184).

## Data Availability

All datasets presented in this study are included in the article. The data used in the analyses described in this article will be available upon reasonable request from the corresponding author.
